# Physical Activity, Sedentary Behavior, and Body Image among Young Adults in Hong Kong: A Cross-Sectional Study

**DOI:** 10.3390/healthcare12181825

**Published:** 2024-09-12

**Authors:** Maria Shuk Yu Hung, Winnie Wing Man Ng, Edward Kwok Yiu Choi

**Affiliations:** 1 S.K. Yee School of Health Sciences, Saint Francis University, Hong Kong, China; 2 School of Nursing, Faculty of Health and Social Sciences, The Hong Kong Polytechnic University, Hong Kong, China; winnie.wm.ng@polyu.edu.hk; 3 School of Education and Languages, Hong Kong Metropolitan University, Hong Kong, China; edwardkyc@gmail.com

**Keywords:** physical activity, sedentary behavior, body image, young adults

## Abstract

The COVID-19 pandemic has considerably impacted the health and lifestyle of various populations worldwide, leading to decreased physical activity, increased sedentary behavior, and increased health problems. This study aimed to investigate Hong Kong young adults’ physical activity, related behaviors, and perceptions of body image during the pandemic. A cross-sectional online survey of young adults aged 18–29 was conducted from February to March 2021 using the well-validated Multidimensional Body Self-Relations Questionnaire (MBSRQ). Among 408 respondents, 52.5% had a normal weight in the BMI range, 45.1% engaged in ≥8 h of sedentary behavior daily, 77.2% engaged in no regular or inadequate physical activity (<2.5 h/week), and only 22.3% joined a fitness club. BMI, regular physical activity, and joining any fitness club/class were significantly associated with the most factors or additional subscales. A multiple linear regression model showed that the underweight respondents (reference group: normal) (β = −0.26, 95% CI: −0.41 to −0.12) were less likely to have high scores of fitness orientation. The respondents who engaged in regular physical activity <2.5 h/week (β = 0.42, 95% CI: 0.29 to 0.54), engaged in regular physical activity ≥2.5 h/week (β = 0.99, 95% CI: 0.84 to 1.14) (reference group: no regular physical activity), and joined a fitness club/class (β = 0.32, 95% CI: 0.18 to 0.45) were more likely to have a high score of fitness orientation. Local governments, non-governmental organizations, schools, and community centers should establish appropriate strategies and activities in order to sufficiently encourage and support young adults’ physical health and well-being.

## 1. Introduction

Regular physical activity and exercise are essential for maintaining good health and improving overall well-being [1]. Physical activities can contribute to cardiovascular health, muscular strength and endurance, weight management, and mental health. However, the COVID-19 pandemic has severely affected human health and lifestyles worldwide in the past few years. The literature shows adverse impacts on health among various populations, including adolescents and young adults, leading to decreased physical activity, increased sedentary behavior, and body image problems [2,3,4,5].

A systematic review of 66 studies comprising 86,981 participants, including different populations, reported decreased physical activity and increased sedentary behavior during community lockdowns in various countries [6]. Another systematic review of university students’ perception of body image and physical activity found that they had reduced physical activity time and intensity but increased sedentary activity time during the pandemic [3]. Furthermore, females were more physically active than males. Similarly, a Chilean study of 469 university students found that sedentary lifestyles and a lack of physical activity negatively affected students’ subjective well-being and general mental health during the pandemic [4]. Despite the pandemic, the World Health Organization emphasized the importance of maintaining sufficient physical activity levels and decreasing sedentary behavior to sustain good health [7].

A healthy body image involves accepting and respecting one’s body, regardless of size or shape, and recognizing the importance of overall well-being beyond physical appearance. Body image has become a global health concern and affects different populations, especially adolescents and young adults [8]. A previous European study investigated 328 normal-weight French male university students who said they were concerned about their body image. The majority (85%) were unsatisfied with their muscularity, which may cause higher risks of depression and eating disorders [9]. An evaluation study of Greek fitness center members showed significant differences in body image among those with different BMI self-classifications (normal, overweight, and obese) [10].

In Asia, the perception of being overweight is more prevalent in South Korea than in Taiwan [11]. Women are more susceptible to societal pressures to maintain a thin body and the ideal body image perception than men [11,12]. Similarly, a Taiwanese study of 2095 adult males’ and females’ perceptions of body shape found that age and gender influenced the respondents’ views on body size [13]. Females were more likely to misperceive themselves as overweight than males. Additionally, males were more likely to misperceive themselves as underweight than females. A local study examined 358 Hong Kong Chinese senior secondary school girls’ body image and eating attitudes, finding that they desired slimness even though only a minority (4.8%) were overweight [14]. Interventions are recommended to improve distorted body image perception [11].

However, the relationships between body image, the significance of physical activity and sedentary behavior, and the concept of health and illness are complex and interconnected, especially under the implementation of city lockdowns and social distancing measures during the pandemic [2,3,6,15,16,17,18]. Goh et al. (2023) systematically reviewed 16 quantitative and 12 qualitative studies from various countries [16]. They reported a comparatively constant association between individuals who expressed higher or lower satisfaction with their bodies and physical appearance. Individuals more satisfied with their body parts were inclined to participate in health checks or screening behaviors. In contrast, those dissatisfied with their body image were less likely to engage in health-related activities [16]. An Iranian study of 535 female high school students conducted in 2020 found significant positive correlations between physical activity levels and body image [2]. A Kosovo study investigated the impact of lockdown measures on the population before and during the pandemic. It reported significantly reduced physical activity in youth (≤25 years), young adults (25 < years ≤ 35), males, overweight individuals (BMI > 25), respondents who resided in urban areas, and young people who were increasingly sedentary during home confinement [17]. Additionally, Runacres et al. (2021) reviewed and analyzed 40 studies concerning the pandemic’s impact on sedentary time and behaviors. They found that children and adults were more undesirably influenced than older adults [18].

The WHO also highlighted the importance of maintaining physical activity and reducing sedentary behavior at home during the pandemic to decrease the risk of severe COVID-19 outcomes [7]. Many public health policies and strategies were implemented worldwide, including lockdowns and restricting access to public areas for physical activity. Likewise, the Hong Kong government also enacted stringent social distancing measures to minimize cross-infection to prevent morbidity and mortality. Hong Kong is a small (1114.57 km^2^) urban city densely populated with about 7.54 million citizens [19]. The average living space per person is also small, at ~13.6 m^2^ [20]. Like other metropolitan cities with efficient transportation and sedentary lifestyles, physical inactivity in Hong Kong is not uncommon [21]. The pandemic further exacerbated the problem due to the closure of public/private recreation areas and premises for sports/physical facilities. The local citizens’ opportunities to access safe indoor/outdoor spaces for physical activity were significantly reduced, particularly for those unable to engage in physical activity at home, adversely affecting the general public’s physical health. Indeed, insufficient physical activity can increase the risk of developing diabetes mellitus, obesity, hypertension, heart disease, cerebrovascular disease, and various forms of cancer [22].

Local studies found that Hong Kong citizens extensively reduced their walking frequencies and sports activities but increased home-cooked meals during the pandemic [15]. Additionally, secondary school students’ physical activity (moderate to vigorous exercise) significantly decreased [23], and local primary school children’s physical fitness and body mass index (BMI) were negatively impacted [24]. However, the impact of public health policies and social distancing measures on local young adults’ body image, physical activity, and sedentary lifestyle remains unclear. A comprehensive understanding of the impacts on young adults’ physical activity and related behaviors is essential; it could assist in establishing appropriate public health strategies and programs to promote regular physical activity habits and minimize sedentary behaviors in the future or in similar lockdown situations. Thus, this study aimed to investigate young adults’ physical activity, related behaviors, and perceptions of body image during the COVID-19 pandemic in Hong Kong.

## 2. Materials and Methods

### 2.1. Study Design

This study adopted a quantitative cross-sectional survey design. This is an easy and inexpensive design used to examine population data at a specific time [25]. It is commonly used to assess factors affecting health, health-related outcomes, and population characteristics, making it suitable for this study.

### 2.2. Subjects and Sampling

People under 18 or over 30 were excluded. The subjects were local young adults aged 18–29 who could understand English and were eligible for participation. Potential subjects were invited to participate, using purposive and snowball sampling methods. The questionnaire was first shared with participants aged 18–29 by using Google forms, and then they were encouraged to share it with their friends aged 18–29. The sample size estimation was based on Solvin’s formula [26], with a known population size and an estimated margin of error. As the population size of the age group 18–29 was 1,002,800 [27,28], and the margin of error was 0.05, a sample size of no less than 400 was required. The following formula was used to calculate the sample size:

*n* = *N*/(1 + *Ne*^2^).

Here, *n* is the sample size, *N* is the population size (*N* = 1,002,800), and *e* is the margin of error (if 5%, *e* = 0.05).

### 2.3. Instrument and Outcome Measures

The self-completed questionnaire was composed of two parts: the respondents’ demographic characteristics and the ratings of the Multidimensional Body-Self Relations Questionnaire (MBSRQ) [29]. The demographic characteristics of the respondents include information on their age group, gender, weight, height (used to calculate body mass index (BMI) range), education, marital status, employment status, religious affiliation, time spent engaging in sedentary behavior per day, regular physical activity per week, and whether they attend any fitness clubs or classes. The MBSRQ is a self-report inventory for the assessment of body image. Permission to use the well-validated MBSRQ [29] was obtained from the author. The MBSRQ evaluates an individual’s perceptions of their physical self, encompassing evaluative and cognitive–behavioral aspects, including physical appearance, body functionality, and biological well-being. The questionnaire contains 69 questions distributed among seven factor subscales and three additional multi-item subscales. The 7 factor subscales encompass 7 appearance evaluation items, 12 appearance orientation items, 3 fitness evaluation items, 13 fitness orientation items, 6 health evaluation items, 8 health orientation items, and 5 illness orientation items. The 3 additional subscales comprise 9 items on body areas satisfaction, 4 on overweight preoccupation, and 2 on self-classified weight. Responses were rated on a five-point scale, ranging from “strongly agree” to “strongly disagree” for agreement-related items and “very satisfied” to “very dissatisfied” for satisfaction-related items [30]. The ten subscales’ internal consistency varied between 0.70 and 0.91, while test–retest reliability ranged from 0.71 to 0.94 [29].

### 2.4. Ethical Considerations

Ethical approval was obtained and the research study was conducted following the Declaration of Helsinki guidelines. Potential respondents were provided with study information through an online information sheet, including details about the aims, risks, benefits, procedures, and voluntary participation. Implied consent was obtained. If the respondents agreed to participate in the study, they continued to fill out the online questionnaire. The respondents could decline to answer questions or opt out of the survey without repercussions. All data were kept confidential, ensuring the respondents’ anonymity. Access to the data was restricted to research team members only. The electronic database file will be erased from the system once the research findings are published or five years after data collection.

### 2.5. Data Collection and Analysis

From 1 November 2020 to 30 April 2021, the Hong Kong government and local citizens experienced and suffered from the impacts of the fourth wave of the COVID-19 epidemic in Hong Kong [31], with a sudden upsurge in infected cases. Data were collected from February to March 2021 using a self-completed online questionnaire. All questionnaires were treated as anonymous. The questionnaires were uploaded to an online survey system, and the investigators were responsible for downloading the questionnaire data.

A statistical analysis was performed using IBM SPSS Statistics for Windows, version 26.0 (IBM Corp, Armonk, NY, USA). The respondents’ demographic characteristics are summarized with descriptive statistics. An independent-sample *t* test (2 groups comparison) and a one-way ANOVA (>2 groups comparison) were used to compare group differences with continuous demographic variables, which follow a normal distribution. Pearson correlation coefficients were used to investigate the strength of the correlations between two continuous variables. Values between 0.5 and 0.7 in absolute value indicated a moderate correlation; values between 0.3 and 0.5 in absolute value indicated a low correlation; and values between 0 and 0.3 in absolute value indicated a negligible correlation [32].

Multiple linear regression was used to determine the significant demographic variables of the MBSRQ subscale. A stepwise elimination procedure was applied to eliminate the insignificant variables. A *p*-value of <0.05 was considered statistically significant.

## 3. Results

### 3.1. Demographic Information of the Respondents

Overall, 408 young adults participated in and completed the questionnaire. Table 1 presents the demographic information of the respondents. About half of the respondents (*n* = 209, 51.2%) were aged 22–25. The majority (*n* = 225, 55.1%) were female. Over half of the respondents (*n* = 214, 52.5%) had a BMI range of normal weight, with 18.6% being underweight (*n* = 76), 12.0% being overweight (*n* = 49), and 16.9% being obese (*n* = 69), based on the WHO’s recommendations for Asian adults [33]. The Asian BMI range (in kg/m^2^) is as follows: underweight <18.5, normal weight 18.5–22.9, overweight 23–24.9, and obese >25. Most respondents (*n* = 337, 82.6%) had a tertiary education level. About half (*n* = 184, 45.1%) engaged in ≥8 h of sedentary behavior daily. The majority of the respondents (77.2%, *n* = 315) did not engage in regular physical activity (40.9%, *n* = 167) or did so for <2.5 h per week (36.3%, *n* = 148). Only about one-fifth of the respondents (*n* = 91, 22.3%) joined a fitness club or class.

### 3.2. Correlation among All MBSRQ Subscales

Table 2 shows the correlations among all MBSRQ subscales. There are significant positive correlations among the MBSRQ subscales. For example, fitness evaluation is positively correlated with fitness orientation (r = 0.65, *p* < 0.01) and health evaluation (r = 0.45, *p* < 0.01); fitness orientation is positively correlated with health orientation (r = 0.60, *p* < 0.01), health evaluation (r = 0.49, *p* < 0.01), and appearance evaluation (r = 0.38, *p* < 0.01); appearance evaluation is positively correlated with body areas satisfaction (r = 0.59, *p* < 0.01), health orientation (*p* = 0.47, *p* < 0.01), and health evaluation (r = 0.41, *p* < 0.01); and appearance orientation is positively correlated with overweight preoccupation (r = 0.44, *p* < 0.01).

### 3.3. Univariate Analysis between Demographics and MBSRQ Subscales

The MBSRQ subscales were analyzed using demographic variables, as shown in Table 3, Table 4 and Table 5. BMI, regular physical activity, and joining any fitness club or class were significantly associated with most factor subscales or additional subscales. For instance, the BMI range was significantly associated with different factor subscales, including appearance evaluation (*p* < 0.001), appearance orientation (*p* < 0.001), fitness evaluation (*p* = 0.004), fitness orientation (*p* = 0.001), health evaluation (*p* = 0.03), and self-classified weight (*p* < 0.001).

### 3.4. Multiple Regression Models for MBSRQ Subscales

Multiple linear regression was applied to identify the significant demographic variables of each MBSRQ subscale. Regarding the outcome of fitness orientation, the model showed that the respondents who were underweight (reference group: normal) (β = −0.26, 95% CI: −0.41 to −0.12) were less likely to have higher scores of fitness orientation. The respondents who engaged in regular physical activity for less than 2.5 h per week (β = 0.42, 95% CI: 0.29 to 0.54), engaged in regular physical activity for more than 2.5 h per week (β = 0.99, 95% CI: 0.84 to 1.14) (reference group: no regular physical activity), and joined a fitness club or class (β = 0.32, 95% CI: 0.18 to 0.45) were more likely to have a higher score of fitness orientation. The adjusted R^2^ of the model was 0.42; thus, it was able to explain 42% of the variation in the outcome score of the significant variables. For the results of other subscales, please refer to Table 6, Table 7 and Table 8 for details.

## 4. Discussion

During the COVID-19 pandemic, people’s health and lifestyle were inevitably affected worldwide. Due to the prolonged pandemic, the Hong Kong government extended social distancing measures for months to prevent cross-infection. These measures included universal mask-wearing, the prohibition of group gatherings, school closures, indoor and outdoor sports premises closures, restrictions on catering businesses’ opening hours, stay-at-home advice, etc. [34]. Hospital and institutional confinement of infected and close-contact cases was implemented [31]. Additionally, the Hong Kong government and some companies implemented work-from-home (WFH) strategies from time to time. These measures also affected Hong Kong residents’ health and daily lifestyles, consistent with other local studies involving various age groups and populations [15,23,24,35,36,37].

The World Health Organization (WHO) recommends that adults aged 18–64 perform at least 300 or 150 min of moderate- or vigorous-intensity aerobic physical activity per week, respectively, to achieve health benefits [7]. Regular physical activity reduces the risk of chronic diseases and enhances cardiopulmonary function [22]. Based on the WHO statistics [38], 80% of adolescents and 31% of adults worldwide could not accomplish the recommended level of physical activity during the pandemic [38]. In Hong Kong, the Health Behavior Survey of 5903 Hong Kong citizens aged 15 or above reported that 16.8% of respondents (15.2% of males and 18.3% of females) had insufficient physical activity before the pandemic [39]. Unsurprisingly, a noticeable increase of 24.8% of citizen respondents (including 22.8% of males and 26.5% of females) had inadequate physical activity during the pandemic [22]. As inactive individuals face a 20 to 30% higher mortality risk than those who are fully active [38], strategies are required to encourage citizens to increase physical activity and exercise daily.

By comparing the WHO’s recommendations with our study data collected in 2021, it was found that most young adult respondents (77.2%, *n* = 315) did not engage in regular physical activity (40.9%, *n* = 167) or did so for <2.5 h per week (36.3%, *n* = 148), and only 22.3% (*n* = 91) joined any fitness club/class. The decrease in physical activity may be attributed to the closure of public and private recreation centers and fitness clubs due to social distancing measures, as noted in other studies [3,6,15]. Indeed, the opportunities for local citizens to access safe indoor/outdoor premises for physical activity were significantly reduced in such a densely populated, small urban city with limited living space, adversely affecting the intention to engage in physical activity. Local citizens considerably reduced their moderate- and high-intensity physical activities and walking exercises [15]. Similarly, another local study conducted in 2020, which involved 631 young adults aged 18–35, reported that only 30% achieved the WHO physical activity guidelines, 57.8% did not engage in vigorous physical activity, and 70% had reduced physical activity [31].

Furthermore, the Health Behaviour Survey of Hong Kong citizens found that 38.9% engaged in ≥8 h of sedentary behavior, such as sitting or reclining at work, at home, or with friends [40]. Our study found that a higher rate (45.1%) of young adults engaged in 8–12 h or >12 h of sedentary behavior during the pandemic. The results are consistent with those of research showing that university students [3] and local young adults [35] increased their sedentary behavior. The lockdown had a more severe impact on children (<18 years) than on young and older adults, leading to an increased sedentary time [18]. This increase in sedentary time negatively affected mental well-being, psychological states, and quality of life. Therefore, there is a need for global and local efforts to establish strategies and promote a reduction in sedentary time.

The Hong Kong Population Health Survey (PHS) conducted from 2020 to 2022 revealed that 54.6% of the general population aged 15–84 were either overweight (22.0%) or obese (32.6%), with women aged 65–84 (57.0%) and men aged 45–54 (74.6%) being more susceptible to obesity and overweight [41]. Among our 408 young adult respondents (aged 18–29), the BMI results showed that 52.5% had a normal weight, 18.6% were underweight, 12.0% were overweight, and 16.9% were obese (based on self-reported weight and height), which was better than the general public.

In our study, we found significant positive correlations among different factor subscales. Compared with the norms of the United States national survey of males and females for the MBSRQ subscales [29,42,43], our study results showed lower mean scores on all the seven factor subscales, except for female illness orientation (3.24 vs. 3.21). This suggests that local young adults may be less satisfied with their body image, physical attractiveness, fitness, and health than individuals in the United States. However, local young female respondents showed a higher alertness to physical illness.

Furthermore, BMI, regular physical activity, and joining any fitness club or class were found to be significantly associated with different factors or additional subscales of the body image construct. Body image refers to how individuals perceive and evaluate their body’s appearance. This perception can influence their thoughts, feelings, and behaviors related to physical activity and fitness [8]. Previous studies found that exercise was associated with a positive body image [44], and individuals who exercised had a more positive body image than those who did not [44]. Additionally, we found that the respondents who engaged in regular physical activity and joined fitness clubs or classes valued fitness and health and were actively involved in activities that enhanced or maintained their fitness and health, while those who had an underweight BMI were not active.

Females are usually more concerned about their weight or body size than males [13] and demonstrate more dissatisfaction regarding their body image [45]. Consistent with previous studies of gender differences in body weight or size [13,46], the young female respondents were more likely to perceive themselves as overweight (35.1%) than males (32.3%), and the male respondents were more likely to perceive themselves as underweight (18.6%) than females (13.4%). Individuals with a self-stigma of being overweight have greater behavioral intention to participate in physical activities in order to control their weight [47]. Additionally, our female respondents focused more on their appearance and were more concerned about body fat, weight vigilance, dieting, and eating habits than males. The results align with those of a study of Korean and Taiwanese individuals conducted by Noh et al. (2018), which observed that females experience more significant societal pressure to be thin than males, impacting body image. Indeed, most women prefer to be slimmer and are likely to overestimate their body weight or size regardless of their actual size [11,12]. In contrast, our male respondents were more confident in their physical fitness, valued their body fitness, and were more concerned about physical activities. Males prefer exercising instead of dieting to alter their body shape, e.g., increasing muscle tone and muscle mass through physical activity [12].

After understanding the impacts on young adults’ physical activity and related behaviors through this study, the results could inform and assist in establishing appropriate public health strategies and programs to promote regular physical activity habits and minimize sedentary behavior in the future or similar emerging infectious diseases with social distancing and institutional or home confinement. For instance, collaboration among stakeholders, such as non-profit organizations, schools, and community centers, could be promoted to establish more recreational activities and behavioral change projects for various populations to integrate physical activity into their daily lives, e.g., regular sports training courses and Physical Activity Week for students, as well as Physical Activity/Sports for ALL campaigns for citizens. Online resources, such as physical activity videos or virtual sports exercises using digital technology to encourage home fitness in future pandemics requiring isolation or social distancing measures, could also be considered.

The study data were gathered using purposive and snowball sampling through online questionnaires filled out by young adults. The sampling methods did not adequately warrant representatives or subject randomization and may raise concerns about bias or generalizability limitations. A future study could consider an appropriate randomized sampling method to minimize potential sampling bias. Although online surveys have several advantages, such as saving time and money when social distancing measures are implemented, the ambiguity of data validity and self-assessment reliability are disadvantages [48]. Although the respondents were encouraged to contact the researchers if questions arose, online self-report questionnaires may lead to reporting bias or a misunderstanding of the questionnaire items. For instance, the BMI was calculated using self-reported data (weight and height) instead of actual measurements.

## 5. Conclusions

The COVID-19 pandemic has considerably impacted the health and lifestyle of various populations worldwide. This study revealed that most Hong Kong young adults were affected in terms of body image, fitness, physical activity, and sedentary behavior. The results indicate that BMI, regular physical activity, and fitness club/class participation were significantly associated with the most factors or additional subscales. The prolonged pandemic, with new public health policies and social distancing measures, worsened the impact on physical activity and lifestyles. Given the physical health benefits of increased physical activity and reduced sedentary behavior, efforts should be made by local governments, non-governmental organizations, schools, and community centers to implement effective public health strategies in order to promote regular physical activity and decrease sedentary behavior among young adults. These activities will contribute to a positive body image, healthier lifestyles, and physical well-being, especially during future public health emergencies requiring isolation or social distancing measures.

## Figures and Tables

**Table 1:** Demographic information of the respondents (total = 408).

	Frequency	Percentage
**Age**		
18–21	107	26.2
22–25	209	51.2
26–29	92	22.5
**Gender**		
Male	183	44.9
Female	225	55.1
**BMI range**		
Underweight	76	18.6
Normal weight	214	52.5
Overweight	49	12.0
Obese	69	16.9
**Educational level**		
Primary	4	1.0
Secondary	67	16.4
Tertiary	337	82.6
**Marital status**		
Single	191	46.8
In a relationship	197	48.3
Married	14	3.4
Cohabitation	6	1.5
**Employment status**		
Working	184	45.1
Student	208	51.0
Unemployed	16	3.9
**Religion**		
Buddhism	14	3.4
Catholicism	3	0.7
Christian	115	28.2
Islam	3	0.7
Taoism	4	1.0
Not applicable	263	64.5
Other	6	1.5
**Hours spent engaged in sedentary behavior per day**		
<8 h	224	54.9
8–12 h	146	35.8
>12 h	38	9.3
**Regular physical activity**		
No	167	40.9
Yes: <2.5 h per week	148	36.3
Yes: ≥2.5 h per week	93	22.8
**Joined any fitness club or class**		
Yes	91	22.3
No	317	77.7

**Table 2:** Correlation among all MBSRQ subscales.

	1	2	3	4	5	6	7	8	9	10
1. Appearance Evaluation	-									
2. Appearance Orientation	0.19 **	-								
3. Fitness Evaluation	0.38 **	0.08	-							
4. Fitness Orientation	0.38 **	0.15 **	0.65 **	-						
5. Health Evaluation	0.41 **	0.08	0.45 **	0.49 **	-					
6. Health Orientation	0.47 **	0.25 **	0.36 **	0.60 **	0.36 **	-				
7. Illness Orientation	0.25 **	0.30 **	0.20 **	0.32 **	0.13 **	0.51 **	-			
8. Body Areas Satisfaction	0.59 **	−0.03	0.31 **	0.31 **	0.27 **	0.38 **	0.17 **	-		
9. Overweight Preoccupation	−0.03	0.44 **	−0.001	0.06	−0.11 *	0.24 **	0.18 **	−0.07	-	
10. Self-Classified Weight	−0.25 **	−0.10 *	0.04	0.01	−0.10 *	−0.004	−0.001	−0.21 **	0.31 **	-

MBSRQ—Multidimensional Body-Self Relations Questionnaire. ** *p* < 0.01, * *p* < 0.05.

**Table 3:** Univariate analysis between demographics and MBSRQ subscales (factor subscales).

		Appearance Evaluation		Appearance Orientation		Fitness Evaluation	
	*n* (%)	Mean (S.D.)	*p* Value	Mean (S.D.)	*p* Value	Mean (S.D.)	*p* Value
**Age**			0.20		0.08		0.21
18–21	107 (26.2)	3.18 (0.61)		3.41 (0.61)		3.26 (0.72)	
22–25	209 (51.2)	3.24 (0.54)		3.51 (0.51)		3.34 (0.72)	
26–29	92 (22.5)	3.10 (0.68)		3.34 (0.72)		3.16 (0.84)	
**Gender**			0.16		* <0.001		* 0.003
Male	183 (44.9)	3.15 (0.62)		3.28 (0.63)		3.40 (0.76)	
Female	225 (55.1)	3.23 (0.57)		3.58 (0.52)		3.18 (0.72)	
**BMI range**			* <0.001		* <0.001		* 0.004
Underweight	76 (18.6)	3.31 (0.61)		3.68 (0.53)		3.05 (0.70)	
Normal weight	214 (52.5)	3.26 (0.55)		3.50 (0.53)		3.36 (0.71)	
Overweight	49 (12.0)	3.15 (0.65)		3.33 (0.50)		3.42 (0.78)	
Obese	69 (16.9)	2.87 (0.53)		3.10 (0.73)		3.16 (0.86)	
**Educational level**			0.14		* 0.05		0.31
Primary/secondary	71 (17.4)	3.10 (0.59)		3.32 (0.58)		3.36 (0.64)	
Tertiary	337 (82.6)	3.21 (0.59)		3.47 (0.59)		3.26 (0.77)	
**Marital status**			0.10		0.23		0.07
Single	191 (46.8)	3.14 (0.53)		3.41 (0.62)		3.24 (0.74)	
In a relationship	197 (48.3)	3.25 (0.62)		3.50 (0.55)		3.36 (0.72)	
Married/cohabitation	20 (4.9)	3.07 (0.73)		3.27 (0.75)		2.90 (1.02)	
**Employment status**			0.51		0.47		0.88
Working	184 (45.1)	3.18 (0.63)		3.43 (0.62)		3.28 (0.80)	
Student	208 (51.0)	3.21 (0.56)		3.47 (0.57)		3.28 (0.70)	
Unemployed	16 (3.9)	3.04 (0.43)		3.31 (0.59)		3.19 (0.72)	
**Religion**			0.11		* 0.004		0.26
Yes	145 (35.5)	3.13 (0.62)		3.56 (0.54)		3.34 (0.73)	
No	263 (64.5)	3.23 (0.57)		3.38 (0.61)		3.25 (0.76)	
**Hours spent engaged in sedentary** **behavior in one day**			0.82		0.16		0.09
<8 h	224 (54.9)	3.18 (0.56)		3.43 (0.51)		3.32 (0.73)	
8–12 h	146 (35.8)	3.20 (0.62)		3.52 (0.62)		3.29 (0.75)	
>12 h	38 (9.3)	3.24 (0.68)		3.28 (0.88)		3.03 (0.83)	
**Regular physical activity**			* <0.001		* 0.02		* <0.001
No	167 (40.9)	3.02 (0.55)		3.35 (0.63)		2.93 (0.64)	
Yes: <2.5 h per week	148 (36.3)	3.25 (0.57)		3.50 (0.55)		3.36 (0.66)	
Yes: ≥2.5 h per week	93 (22.8)	3.40 (0.62)		3.53 (0.57)		3.77 (0.75)	
**Joined any fitness club or class**			* <0.001		0.16		* <0.001
Yes	91 (22.3)	3.51 (0.58)		3.52 (0.57)		3.65 (0.77)	
No	317 (77.7)	3.10 (0.56)		3.42 (0.60)		3.17 (0.71)	

* *p* < 0.05. Continuous variables were analyzed using independent-samples *t* test (2 groups comparison) or one-way ANOVA (>2 groups comparison).

**Table 4:** Univariate analysis between demographics and MBSRQ subscales (factor subscales).

	Fitness Orientation		Health Evaluation		Health Orientation		Illness Orientation	
	Mean (S.D.)	*p* Value	Mean (S.D.)	*p* Value	Mean (S.D.)	*p* Value	Mean (S.D.)	*p* Value
**Age**		0.65		0.99		0.58		* 0.04
18–21	3.26 (0.74)		3.19 (0.54)		3.23 (0.55)		3.27 (0.61)	
22–25	3.20 (0.68)		3.20 (0.56)		3.17 (0.50)		3.20 (0.63)	
26–29	3.16 (0.75)		3.20 (0.53)		3.16 (0.56)		3.05 (0.73)	
**Gender**		* 0.001		0.27		0.27		0.08
Male	3.34 (0.74)		3.23 (0.53)		3.15 (0.59)		3.12 (0.69)	
Female	3.10 (0.67)		3.17 (0.56)		3.21 (0.47)		3.24 (0.61)	
**BMI range**		* 0.001		* 0.03		0.06		0.17
Underweight	2.95 (0.71)		3.17 (0.55)		3.08 (0.51)		3.16 (0.66)	
Normal weight	3.28 (0.69)		3.26 (0.57)		3.24 (0.48)		3.25 (0.65)	
Overweight	3.39 (0.65)		3.22 (0.56)		3.20 (0.57)		3.11 (0.51)	
Obese	3.13 (0.74)		3.04 (0.43)		3.10 (0.62)		3.07 (0.74)	
**Educational level**		0.81		0.98		* 0.02		0.96
Primary/secondary	3.19 (0.66)		3.20 (0.53)		3.05 (0.49)		3.18 (0.57)	
Tertiary	3.21 (0.72)		3.20 (0.55)		3.21 (0.53)		3.19 (0.67)	
**Marital status**		* 0.03		0.65		* 0.001		0.32
Single	3.12 (0.70)		3.19 (0.59)		3.07 (0.53)		3.14 (0.68)	
In a relationship	3.30 (0.70)		3.22 (0.50)		3.26 (0.48)		3.23 (0.60)	
Married/cohabitation	3.16 (0.80)		3.13 (0.59)		3.47 (0.74)		3.21 (0.82)	
**Employment status**		0.18		0.39		* 0.01		0.22
Working	3.23 (0.73)		3.16 (0.57)		3.16 (0.54)		3.16 (0.68)	
Student	3.21 (0.70)		3.24 (0.54)		3.23 (0.50)		3.23 (0.60)	
Unemployed	2.89 (0.60)		3.17 (0.29)		2.82 (0.56)		2.95 (0.89)	
**Religion**		0.23		0.53		0.26		0.21
Yes	3.26 (0.67)		3.22 (0.56)		3.22 (0.48)		3.24 (0.65)	
No	3.18 (0.73)		3.19 (0.54)		3.16 (0.55)		3.16 (0.65)	
**Hours spent engaged in sedentary behavior per day**		0.24		0.64		0.16		0.96
<8 h	3.26 (0.67)		3.21 (0.56)		3.19 (0.49)		3.18 (0.61)	
8–12 h	3.15 (0.72)		3.17 (0.53)		3.13 (0.54)		3.19 (0.65)	
>12 h	3.10 (0.86)		3.25 (0.53)		3.31 (0.67)		3.22 (0.87)	
**Regular physical activity**		* <0.001		* <0.001		* <0.001		* 0.002
No	2.77 (0.51)		3.02 (0.47)		2.96 (0.45)		3.08 (0.60)	
Yes: <2.5 h per week	3.27 (0.60)		3.19 (0.53)		3.22 (0.52)		3.19 (0.67)	
Yes: ≥2.5 h per week	3.90 (0.60)		3.54 (0.56)		3.51 (0.48)		3.38 (0.67)	
**Joined any fitness club** **or class**		* <0.001		* 0.01		* <0.001		* 0.001
Yes	3.72 (0.68)		3.35 (0.61)		3.46 (0.58)		3.38 (0.69)	
No	3.06 (0.65)		3.16 (0.52)		3.10 (0.48)		3.13 (0.63)	

* *p* < 0.05. Continuous variables were analyzed using independent-samples *t* test (2 groups comparison) or one-way ANOVA (>2 groups comparison).

**Table 5:** Univariate analysis between demographics and MBSRQ subscales (additional subscales).

	Body Areas Satisfaction		Overweight Preoccupation		Self-Classified Weight	
	Mean (S.D.)	*p* Value	Mean (S.D.)	*p* Value	Mean (S.D.)	*p* Value
**Age**		* 0.007		0.10		* 0.02
18–21	3.08 (0.69)		2.93 (1.01)		3.00 (0.86)	
22–25	3.13 (0.62)		2.74 (0.85)		3.08 (0.74)	
26–29	2.85 (0.72)		2.95 (0.98)		3.34 (0.89)	
**Gender**		0.36		* <0.001		0.75
Male	3.02 (0.67)		2.60 (0.93)		3.13 (0.86)	
Female	3.08 (0.66)		3.03 (0.88)		3.10 (0.79)	
**BMI range**		0.26		0.23		* <0.001
Underweight	3.05 (0.72)		2.87 (1.16)		2.35 (0.82)	
Normal weight	3.10 (0.59)		2.77 (0.83)		3.05 (0.57)	
Overweight	3.08 (0.70)		3.06 (0.93)		3.48 (0.59)	
Obese	2.89 (0.79)		2.86 (0.92)		3.88 (0.79)	
**Educational level**		0.52		0.70		0.89
Primary/secondary	3.01 (0.58)		2.80 (0.98)		3.13 (0.86)	
Tertiary	3.06 (0.69)		2.85 (0.92)		3.11 (0.81)	
**Marital status**		* 0.001		0.64		0.07
Single	2.96 (0.64)		2.79 (0.90)		3.09 (0.85)	
In a relationship	3.17 (0.67)		2.88 (0.93)		3.09 (0.76)	
Married/cohabitation	2.78 (0.67)		2.86 (1.18)		3.53 (0.92)	
**Employment status**		0.16		0.69		0.09
Working	3.03 (0.72)		2.85 (0.95)		3.20 (0.85)	
Student	3.10 (0.62)		2.85 (0.92)		3.05 (0.78)	
Unemployed	2.79 (0.71)		2.64 (0.82)		2.88 (0.85)	
**Religion**		0.50		* <0.001		0.53
Yes	3.08 (0.73)		3.08 (0.94)		3.15 (0.78)	
No	3.04 (0.63)		2.70 (0.89)		3.10 (0.84)	
**Hours spent engaged in sedentary behavior per day**		0.93		0.49		0.94
<8 h	3.06 (0.65)		2.83 (0.85)		3.11 (0.80)	
8–12 h	3.04 (0.61)		2.79 (0.95)		3.13 (0.80)	
>12 h	3.04 (0.93)		3.05 (1.22)		3.08 (1.00)	
**Regular physical activity**		* 0.002		0.02		0.89
No	2.96 (0.66)		2.68 (0.92)		3.11 (0.92)	
Yes: <2.5 h per week	3.02 (0.63)		2.95 (0.86)		3.13 (0.79)	
Yes: ≥2.5 h per week	3.26 (0.71)		2.95 (1.01)		3.09 (0.66)	
**Joined any fitness club or class**		* <0.001		0.10		0.34
Yes	3.30 (0.74)		2.98 (1.05)		3.19 (0.79)	
No	2.98 (0.63)		2.80 (0.89)		3.09 (0.82)	

* *p* < 0.05. Continuous variables were analyzed using independent-samples *t* test (2 groups comparison) or one-way ANOVA (>2 groups comparison).

**Table 6:** Multiple regression models for MBSRQ subscales (factor subscales).

	Appearance Evaluation		Appearance Orientation		Fitness Evaluation		Fitness Orientation	
	β (95% CI of β)	*p* Value	β (95% CI of β)	*p* Value	β (95% CI of β)	*p* Value	β (95% CI of β)	*p* Value
**Gender**								
Male			−0.20 (−0.31 to −0.08)	* 0.001				
Female			1					
**BMI range**								
Normal weight	1		1		1		1	
Underweight	0.08 (−0.07 to 0.22)	0.30	0.21 (0.06 to 0.35)	* 0.01	−0.25 (−0.43 to −0.08)	* 0.004	−0.26 (−0.41 to −0.12)	* <0.001
Overweight	−0.16 (−0.33 to 0.01)	0.06	−0.12 (−0.29 to 0.06)	0.20	−0.002 (−0.21 to 0.20)	0.99	0.02 (−0.15 to 0.19)	0.82
Obese	−0.41 (−0.56 to −0.26)	* <0.001	−0.29 (−0.45 to −0.14)	* <0.001	−0.10 (−0.28 to 0.08)	0.26	−0.10 (−0.25 to 0.05)	0.17
**Educational level**								
Primary/secondary			−0.18 (−0.32 to −0.03)	* 0.02				
Tertiary			1					
**Marital status**								
Single					1			
In a relationship					0.05 (−0.08 to 0.18)	0.49		
Married/cohabitation					−0.47 (−0.77 to −0.16)	* 0.003		
**Religion**								
Yes	−0.13 (−0.24 to −0.02)	* 0.03	0.19 (0.08 to 0.30)	* 0.001				
No	1		1					
**Hours spent engaged in sedentary behavior per day**								
≤12 h			1		1			
>12 h			−0.21 (−0.40 to −0.03)	0.02	−0.27 (−0.49 to −0.05)	* 0.02		
**Regular physical activity**								
No	1				1		1	
Yes: <2.5 h per week	0.13 (0.01 to 0.25)	* 0.04			0.34 (0.19 to 0.49)	* <0.001	0.42 (0.29 to 0.54)	* <0.001
Yes: ≥2.5 h per week	0.26 (0.11 to 0.41)	* 0.001			0.75 (0.57 to 0.93)	* < 0.001	0.99 (0.84 to 1.14)	* <0.001
**Joined any fitness club or class**								
Yes	0.35 (0.22 to 0.49)	* <0.001	0.16 (0.03 to 0.29)	0.01	0.24 (0.08 to 0.40)	* 0.004	0.32 (0.18 to 0.45)	* <0.001
No	1		1		1		1	
	Adjusted R^2^ = 0.18		Adjusted R^2^ = 0.16		Adjusted R^2^ = 0.24		Adjusted R^2^ = 0.42	

* *p* < 0.05. Multiple regression was conducted, and stepwise elimination procedure was applied.

**Table 7:** Multiple regression models for MBSRQ subscales (factor subscales).

	Health Evaluation		Health Orientation		Illness Orientation	
	β (95% CI of β)	*p* Value	β (95% CI of β)	*p* Value	β (95% CI of β)	*p* Value
**Age**						
18–21					1	
22–25					−0.05 (−0.20 to 0.10)	0.50
26–29					−0.21 (−0.39 to −0.03)	* 0.02
**Gender**						
Male			−0.14 (−0.24 to −0.04)	* 0.005	−0.15 (−0.27 to −0.02)	* 0.02
Female			1		1	
**BMI range**						
Normal weight	1		1			
Underweight	−0.07 (−0.21 to 0.06)	0.27	−0.15 (−0.27 to −0.03)	* 0.02		
Overweight	−0.07 (−0.23 to 0.09)	0.38	−0.01 (−0.16 to 0.13)	0.86		
Obese	−0.20 (−0.34 to −0.06)	* 0.01	−0.05 (−0.18 to 0.08)	0.46		
**Educational level**						
Primary/secondary			−0.14 (−0.26 to −0.02)	* 0.03		
Tertiary			1			
**Marital status**						
Single			1			
In a relationship			0.13 (0.03 to 0.22)	* 0.009		
Married/cohabitation			0.37 (0.15 to 0.59)	* <0.001		
**Employment status**						
Working			1			
Student			0.15 (0.05 to 0.24)	*0.003		
Unemployed			−0.17 (−0.41 to 0.08)	0.18		
**Regular physical activity**						
No	1		1		1	
Yes: <2.5 h per week	0.15 (0.04 to 0.27)	* 0.009	0.24 (0.13 to 0.34)	* <0.001	0.07 (−0.07 to 0.22)	0.31
Yes: ≥2.5 h per week	0.51 (0.38 to 0.64)	* <0.001	0.49 (0.36 to 0.62)	* <0.001	0.25 (0.08 to 0.43)	* 0.005
**Joined any fitness club or class**						
Yes			0.18 (0.06 to 0.29)	* 0.003	0.20 (0.04 to 0.36)	* 0.02
No			1		1	
	Adjusted R^2^ = 0.14		Adjusted R^2^ = 0.25		Adjusted R^2^ = 0.06	

* *p* < 0.05. Multiple regression was conducted, and stepwise elimination procedure was applied.

**Table 8:** Multiple regression models for MBSRQ subscales (additional subscales).

	Body Areas Satisfaction		Overweight Preoccupation		Self-Classified Weight	
	β (95% CI of β)	*p* Value	β (95% CI of β)	*p* Value	β (95% CI of β)	*p* Value
**Age**						
18–21	1					
22–25	0.06 (−0.09 to 0.21)	0.45				
26–29	−0.20 (−0.38 to −0.01)	* 0.04				
**Gender**						
Male			−0.57 (−0.76 to −0.39)	* <0.001	−0.36 (−0.50 to −0.23)	* <0.001
Female			1		1	
**BMI range**						
Normal weight			1		1	
Underweight			0.07 (−0.16 to 0.30)	0.53	−0.76 (−0.93 to −0.59)	* <0.001
Overweight			0.45 (0.17 to 0.72)	* 0.001	0.53 (0.33 to 0.74)	* <0.001
Obese			0.40 (0.15 to 0.64)	* 0.002	0.97 (0.78 to 1.15)	* <0.001
**Marital status**						
Single	1					
In a relationship	0.16 (0.03 to 0.29)	* 0.02				
Married/cohabitation	−0.14 (−0.45 to 0.18)	0.39				
**Religion**						
Yes			0.33 (0.16 to 0.51)	* <0.001		
No			1			
**Regular physical activity**						
No			1			
Yes: <2.5 h per week	−0.01 (−0.16 to 0.13)	0.85	0.32 (0.13 to 0.52)	* 0.001		
Yes: ≥2.5 h per week	0.20 (0.03 to 0.38)	* 0.02	0.32 (0.10 to 0.55)	* 0.005		
**Joined any fitness club or class**						
Yes	0.25 (0.09 to 0.41)	* 0.002				
No	1					
	Adjusted R^2^ = 0.09		Adjusted R^2^ = 0.13		Adjusted R^2^ = 0.38	

* *p* < 0.05. Multiple regression was conducted, and stepwise elimination procedure was applied.

## Data Availability

The data presented in this study are available on reasonable request from the corresponding author.
